# Investigating age and regional effects on the relation between the incidence of heat-related ambulance transport and daily maximum temperature or WBGT

**DOI:** 10.1186/s12199-021-01034-z

**Published:** 2021-12-10

**Authors:** Satoru Ueno, Daisuke Hayano, Eiichi Noguchi, Tohru Aruga

**Affiliations:** 1 Work Environment Research Group, National Institute of Occupational Safety and Health, Japan Organization of Occupational Health and Safety, Kawasaki, Japan; 2 Department of Emergency and Critical Care Medicine, Kanto Rosai Hospital, Japan Organization of Occupational Health and Safety, Kawasaki, Japan; 3 Yokohama Branch, General Incorporated Association Toda Medical Group Headquarters, Yokohama, Japan; 4 Japan Organization of Occuational Health and Safety, Kawasaki, Japan

**Keywords:** Heat-related ambulance transport, Age, Region, Japan

## Abstract

**Background:**

Although age and regional climate are considered to have effects on the incidence ratio of heat-related illness, quantitative estimation of age or region on the effect of occurring temperature for heat stroke is limited.

**Methods:**

By utilizing data on the number of daily heat-related ambulance transport (HAT) in each of three age groups (7–17, 18–64, 65 years old, or older) and 47 prefectures in Japan, and daily maximum temperature (DMT) or Wet Bulb Globe Temperature (DMW) of each prefecture for the summer season, the effects of age and region on heat-related illness were studied. Two-way ANOVA was used to analyze the significance of the effect of age and 10 regions in Japan on HAT. The population-weighted average of DMT or DMW measured at weather stations in each prefecture was used as DMT or DMW for each prefecture. DMT or DMW when HAT is one in 100,000 people (T_1_ and W_1_, respectively) was calculated for each age category and prefecture as an indicator of heat acclimatization. The relation between T_1_ or W_1_ and average DMT or DMW of each age category and prefecture were also analyzed.

**Results:**

HAT of each age category and prefecture was plotted nearly on the exponential function of corresponding DMT or DMW. Average *R*^2^ of the regression function in 47 prefectures in terms of DMW was 0.86, 0.93, and 0.94 for juveniles, adults, and elderly, respectively. The largest regional difference of W_1_ in 47 prefectures was 4.5 and 4.8 °C for juveniles and adults, respectively between Hokkaido and Tokyo, 3.9 °C for elderly between Hokkaido and Okinawa. Estimated W_1_ and average DMT or DMW during the summer season for 47 prefectures was linearly related. Regarding age difference, the regression line showed that W_1_ of the prefecture for DMW at 30 °C of WBGT was 31.1 °C, 32.4 °C, and 29.8 °C for juveniles, adults, and elderly, respectively.

**Conclusions:**

Age and regional differences affected the incidence of HAT. Thus, it is recommended that public prevention measures for heat-related disorders take into consideration age and regional variability.

## Background

A large number of heat-related mortalities (HRM) and illnesses (HRI) occur during extreme summer weather [1, 2]. Currently, the annual number of HRM cases in Japan is over 1000 in a hot summer [3]. As greenhouse gas concentrations continue to increase, the annual average temperature of Japan is estimated to increase by 4.5 °C by the end of this century unless appropriate measures are taken [4]. Previous studies have shown that HRI increases sharply when daily maximum temperature (DMT) exceeds 32 °C [5] and HRM have a strong relationship with the number of days when DMT is over 35 °C [6]. Previous research [2, 7, 8] also found that regional climatic characteristics influence the frequency of HRM. Mortality impact due to heat waves was larger in the north than in the south of the USA [2, 7]. In high latitude areas of the USA, emergency departures with hyperthermia occurred at a lower DMT than in low latitude area [8]. In areas where the summer weather is mild, acclimatization to high temperatures does not occur sufficiently. Consequently, many people suffer from HRI when the weather suddenly heats up.

Since Japan extends from the subarctic zone of Hokkaido in the north to the subtropical zone of Okinawa in the south, the incidence of HRM or HRI is expected to differ for each region. HRM is reported to be higher on the Sea of Japan side than the Pacific side and regional summer heat stress affects prefectural HRM [9]. Yokoyama [10] showed a relationship between DMT and frequency of HRI in major cities. However, quantitative regional characteristic differences regarding the relationship between temperature and frequency of HRI have not been studied.

Aging is also one of the influencing factors affecting the incidence of HRM or HRI [11]. To prevent an increase in core temperature in a hot environment, humans increase skin blood flow and sweat rate to dissipate heat from the body. These responses put a great burden on the cardiovascular system to increase cardiac output. Since older people have lower cardiac output, sweat rate, and plasma volume, an adequate thermoregulatory response during extreme hot weather can be challenging for older people. In Japan, the percentage of elderly aged 65 or older increased from 7.1% in 1970 to 28.8% in 2020 [12]. The number of deaths due to heat stroke in the vital statistics released by the Ministry of Health, Labor and Welfare reached a record high of 1731 persons in 2010, of which 79.3% were 65 years old or older. The mortality rate due to heat stroke showed a higher value associated with age [3].

HRI has a serious impact on society because it causes death or severe sequelae if appropriate measures are not taken. Although there are many kinds of criteria for heat stroke which are referred to in countermeasures for HRI, they do not consider regional and age differences in HRI. Thus, in order to plan an effective HRI mitigation strategy, it is necessary to have a better quantitative understanding of the incidence of HRI caused by regional or age differences. We investigated the relationship between HRI in 47 prefectures in Japan and DMT in each prefecture. We tested our hypothesis that the onset temperature of HRI is higher in a region where the summer weather is typically hot than mild and that HRI occurs at a relatively lower temperature in the elderly.

## Methods

### Data collection

Daily maximum temperature (DMT), daily maximum Wet Bulb Globe Temperature (DMW), and daily number of heat-related ambulance transport (HAT) for six age categories from 2017 to 2020 were collected for all 47 prefectures of Japan. Individuals cannot be identified from the data. The six age categories were the following: newborn (less than 28 days old), infants (28 days to 6 years old), juveniles (7 to 17 years old), adults (18 to 64 years old), elderly (65 years old or older), and unknown. Since the sum of the age categories for unknown, newborns, and infants constitute only 0.7% of the total, we analyzed the other three age groups: juveniles (10.7%), adults (36.2%), and elderly (52.3%). The number of HAT was obtained from the website of the Fire and Disaster Management Agency of the Ministry of Internal Affairs and Communications. Since a previous study [13] has pointed out that the relationship between weather and HRM was different depending on the season, we extracted 178,357 incidents that occurred from July 21st to the end of August for each year from 2017 to 2020, which is defined as “T period” in this analysis. The DMT or DMW value of each of the 47 prefectures were estimated by the average of DMT measured at three or six meteorological stations in each prefecture, which was weighted by the population of the city where the meteorological station is situated. In Hokkaido, which has the largest area of 47 prefectures, the average DMT differed greatly in each city during the period covered by this study. Therefore, to calculate the DMT of Hokkaido, the DMT of the six largest cities in Hokkaido were averaged by weighting its population. Meanwhile, for the other prefectures, we adopted three cities with a meteorological observatory in descending order of its population in the prefecture. If the population of the city is similar, we adopted cities where the cities in the prefecture would be scattered as much as possible. DMT of the three cities were averaged by weighting their population to obtain the DMT of the prefecture. The DMT of each city was downloaded from the website of the Japan Meteorological Agency [14]. The population of each city and prefecture was estimated by using data from the Statistics Bureau of Japan [15] as of October 2019. Similarly, the DMW values of each of the 47 prefectures were calculated. The estimated value (including observed values) of WBGT were downloaded from the website of the Ministry of the Environment [16], which provides data for about 850 points nationwide. In the eight largest cities, actual WBGT was measured, but in other places WBGT values were obtained from the following prediction formulas [17].

1 $$\mathrm{WBGT}=0.735\times {\mathrm{T}}_{\mathrm{a}}+0.374\times \mathrm{RH}+0.00292\times {\mathrm{T}}_{\mathrm{a}}\times \mathrm{RH}+7.619\times \mathrm{SR}-4.557\times {\mathrm{SR}}^2-0.0572\times \mathrm{WS}-4.064$$

In this equation, *T*_a_ is dry bulb temperature (°C), RH is relative humidity (%), SR is global solar radiation (kW/m^2^), and WS is wind speed (m/s). Since the published WBGT data is an hourly value, the highest value of 24 WBGT readings in a day was regarded as the DMW. While temperature is easy to measure, it is a rough heat index when estimating the effect of a heat environment on the human body. WBGT is a more sophisticated heat index, but laborious to measure accurately. Since the two indices have their own advantages, HAT was analyzed using the two indices.

**Fig. 1:**
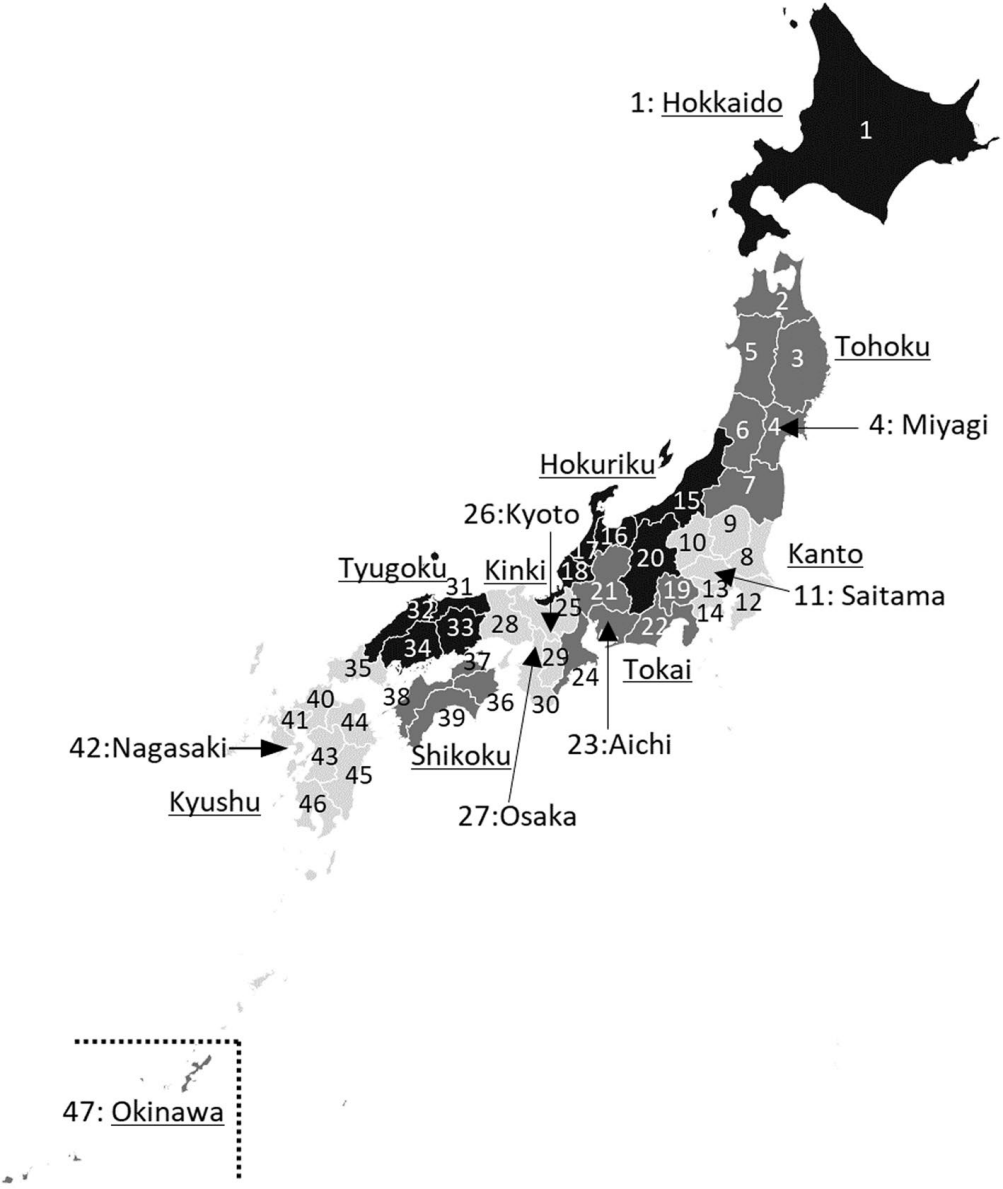
Location of 10 regions and 47 prefectures in Japan. The names of 10 regions are underlined. The number of prefectures in Figure 1 corresponds to the number of prefectures in Table 1

The incidence of HAT was calculated for 3 age classes and 10 regions (Fig. 1) into which 47 prefectures were grouped and tested for a significant difference by age and region by using two-way ANOVA (STATA15, Stata Corp.) to obtain a general trend. The 10 regions are Hokkaido, Tohoku, Kanto, Hokuriku, Tokai, Kinki, Tyugoku, Shikoku, Kyushu, and Okinawa. We mainly followed the meteorological classification by the Japan Meteorological Agency. Southern Kyushu was integrated with Northern Kyushu because Southern Kyushu consists of only two prefectures. Nagano was integrated into Hokuriku, and Yamanashi was integrated into Tokai.

**Table 1:** Mean, Q3, and maximum of DMT and HAT incidence of 7–17, 18–64, 65 years old, and all ages for 47 prefectures and 10 regions

Region	No.	Prefecture	Daily maximum temperature			Heat Ambulance Transport incidence			
			mean	Q3	max	7-17	18-64	65-	all
Hokkaido	1	Hokkaido	26.1	28.3	33.3	0.40	0.22	0.59	0.34
Tohoku	2	Aomori	28.0	30.4	35.5	0.66	0.25	0.81	0.47
	3	Iwate	28.7	31.9	35.2	0.73	0.39	1.31	0.71
	4	Miyagi	28.5	31.3	37.0	0.91	0.46	1.31	0.72
	5	Akita	29.9	31.8	37.6	0.94	0.47	1.28	0.79
	6	Yamagata	30.8	33.7	38.5	1.12	0.48	1.43	0.84
	7	Fukushima	29.7	32.2	34.7	1.08	0.60	1.79	1.00
Kanto	8	Ibaraki	30.4	32.9	36.6	1.02	0.64	1.48	0.90
	9	Tochigi	31.6	34.6	37.7	1.15	0.56	1.45	0.85
	10	Gunma	32.8	36.0	40.1	1.54	0.62	1.84	1.05
	11	Saitama	32.4	35.6	39.6	0.94	0.56	1.69	0.88
	12	Chiba	31.6	33.5	37.2	0.93	0.52	1.37	0.77
	13	Tokyo	32.1	34.5	39.0	0.60	0.42	1.51	0.67
	14	Kanagawa	31.8	34.1	37.1	0.70	0.40	1.16	0.61
Hokuriku	15	Niigata	31.2	33.4	39.6	0.87	0.62	1.50	0.90
	16	Toyama	32.0	34.2	38.5	0.79	0.40	1.23	0.69
	17	Ishikawa	32.0	34.1	37.6	1.06	0.55	1.59	0.89
	18	Fukui	32.8	34.9	37.3	0.96	0.55	1.51	0.87
	20	Nagano	31.9	34.5	37.3	0.83	0.41	1.29	0.72
Tokai	19	Yamanashi	33.6	36.0	39.8	1.43	0.52	1.62	0.93
	21	Gifu	34.1	36.5	39.2	1.25	0.62	1.80	1.01
	22	Shizuoka	32.3	33.5	37.6	0.95	0.55	1.42	0.83
	23	Aichi	34.0	36.0	39.9	0.89	0.62	1.82	0.92
	24	Mie	32.3	33.9	37.8	1.38	0.72	1.82	1.08
Kinki	25	Shiga	32.9	35.1	37.0	1.08	0.50	1.39	0.77
	26	Kyoto	34.4	36.5	39.3	1.16	0.61	1.98	1.03
	27	Osaka	34.2	35.6	38.3	1.04	0.58	1.65	0.90
	28	Hyogo	32.9	34.6	37.3	1.04	0.55	1.77	0.93
	29	Nara	33.8	35.5	37.8	1.80	0.71	1.73	1.11
	30	Wakayama	33.1	34.6	37.4	1.37	0.78	1.72	1.12
Tyugoku	31	Tottori	33.1	35.3	37.9	1.49	0.68	2.19	1.22
	32	Shimane	32.3	34.2	38.0	1.25	0.60	1.71	1.01
	33	Okayama	33.5	35.0	37.2	1.10	0.75	2.19	1.19
	34	Hiroshima	33.2	35.2	37.1	0.78	0.58	1.84	0.94
Shikoku	36	Tokushima	32.6	33.9	37.2	1.36	0.58	1.75	1.02
	37	Kagawa	32.6	34.0	37.3	0.98	0.65	1.90	1.05
	38	Ehime	33.2	34.6	36.6	1.19	0.67	1.82	1.07
	39	Kochi	32.7	33.9	37.8	1.40	0.79	1.95	1.22
Kyushu	35	Yamaguchi	32.8	34.7	36.4	1.00	0.56	1.31	0.83
	40	Fukuoka	33.3	34.9	36.9	0.96	0.57	1.57	0.86
	41	Saga	33.1	34.9	37.3	1.70	0.78	1.65	1.10
	42	Nagasaki	32.5	34.0	37.5	1.11	0.69	1.84	1.08
	43	Kumamoto	33.7	35.7	37.8	1.40	0.79	1.96	1.18
	44	Oita	32.8	34.2	36.7	1.05	0.64	1.86	1.05
	45	Miyazaki	32.3	33.7	36.3	1.69	0.69	1.70	1.09
	46	Kagoshima	33.2	34.4	36.8	1.48	0.83	2.15	1.27
Okinawa	47	Okinawa	32.3	33.2	34.8	0.92	0.53	1.16	0.69
All prefectures			32.5	34.0	37.5	0.96	0.54	1.55	0.84
Hokkaido			26.1	28.3	33.3	0.40	0.22	0.59	0.34
Tohoku			29.2	31.9	34.7	0.92	0.45	1.35	0.76
Kanto			31.9	34.4	38.1	0.83	0.48	1.46	0.74
Hokuriku			31.8	34.1	36.7	0.89	0.51	1.41	0.81
Tokai			33.4	35.1	38.5	1.03	0.61	1.71	0.93
Kinki			33.7	35.3	37.8	1.12	0.59	1.72	0.94
Tyugoku			33.2	35.1	36.6	1.00	0.65	1.97	1.06
Shikoku			32.8	34.1	36.5	1.20	0.67	1.85	1.08
Kyushu			33.1	34.6	36.1	1.20	0.66	1.73	1.01
Okinawa			32.3	33.2	34.8	0.92	0.53	1.16	0.69

**Fig. 2:**
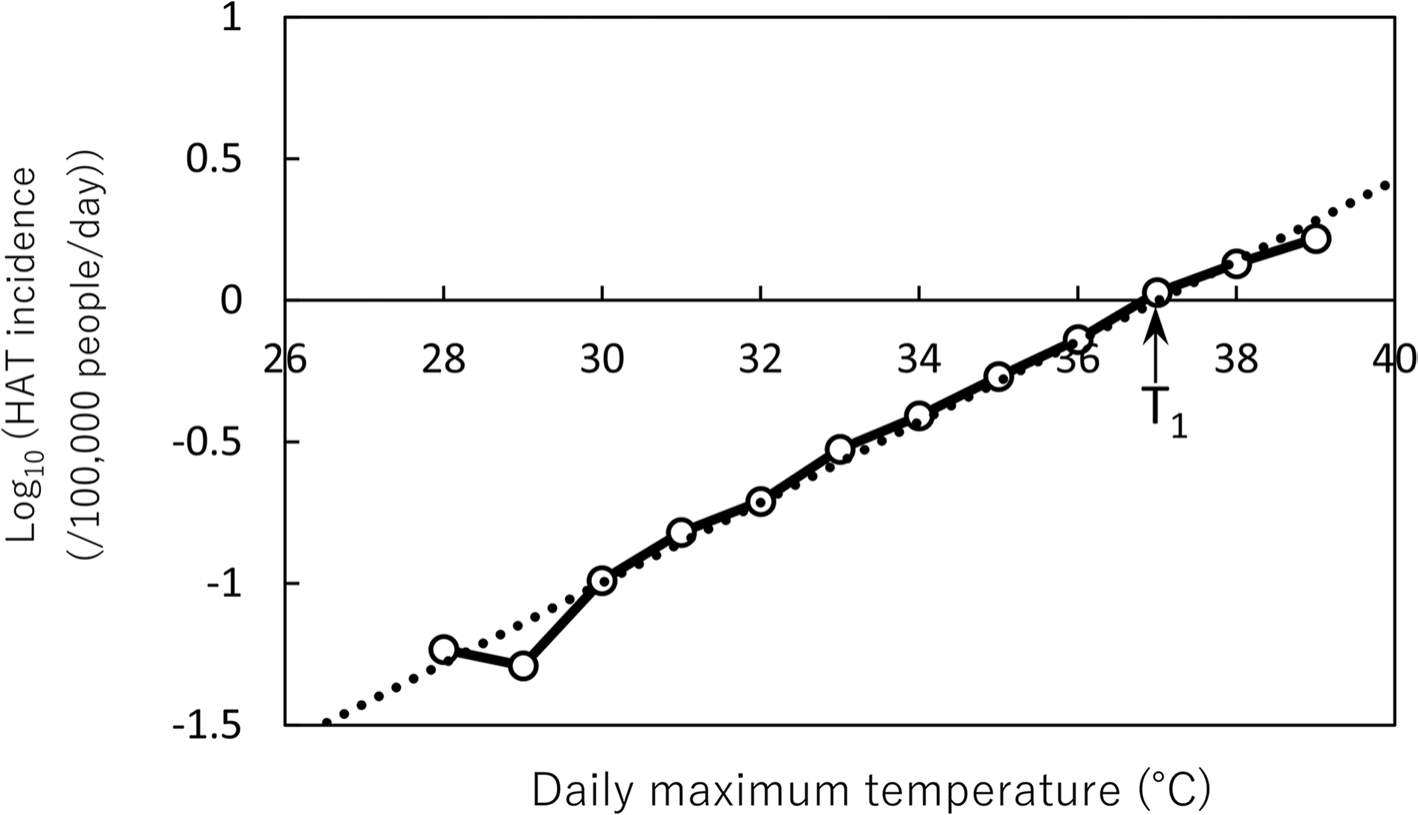
Relation between HAT incidence and DMT. *HAT* heat-related ambulance transport, *DMT* daily maximum temperature, T_1_ is DMT at the intersection of the regression line and *y* = 0 (1 in 100,000 people). The displayed data is from Kyoto for adults

More detailed analysis was conducted for each of the 47 prefectures. In order to quantitatively determine the relationship between weather conditions and the onset of heat stroke, DMT (denoted as T_1_) and DMW (denoted as W_1_) when HAT is expected to occur at a rate of one in 100,000 people per day were calculated by using the linear regression line between the logarithm of the heat stroke incidence per day and the DMT and DMW (Fig. 2). Since a previous study [18] pointed out that HAT incidence in Japan could be expressed as an exponential function of daily maximum temperature, we used the function in the analysis. T_1_ and W_1_ were used as an index of heat acclimatization of local people. Microsoft Excel 2019 was used to display the data link between the daily number of HAT and DMT or DMW, and the calculation of T_1_ and W_1_ for each prefecture.

## Results

**Fig. 3:**
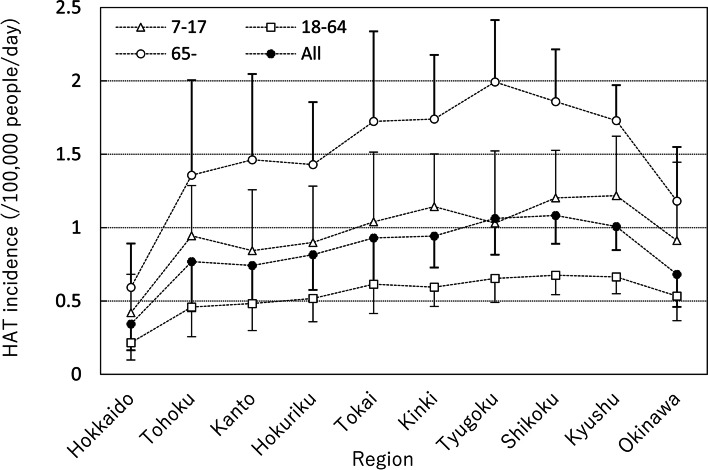
HAT incidence by age group and region. *HAT* heat-related ambulance transport. The error bar indicates the standard deviation of average HAT for each year from 2017 to 2020. If the error bars are shown on both sides, they will overlap and it will be difficult to distinguish them, then only one side is shown

Annual incidences per 1000 persons of HAT from July 21st to the end of August in Japan were 0.41, 0.23, and 0.65 for juveniles, adults, and elderly, respectively. Figure 3 shows the number of HAT per 100,000 people per day of 10 regions for 3 age groups. In each region, the incidence of heat stroke was highest in the elderly, followed by juveniles and adults. Hokkaido was the lowest of 10 regions. Incidence tended to increase in the south but decreased in the southernmost island prefecture of Okinawa. In two-way ANOVA, both region and age group significantly affected the incidence of HAT.

**Fig. 4:**
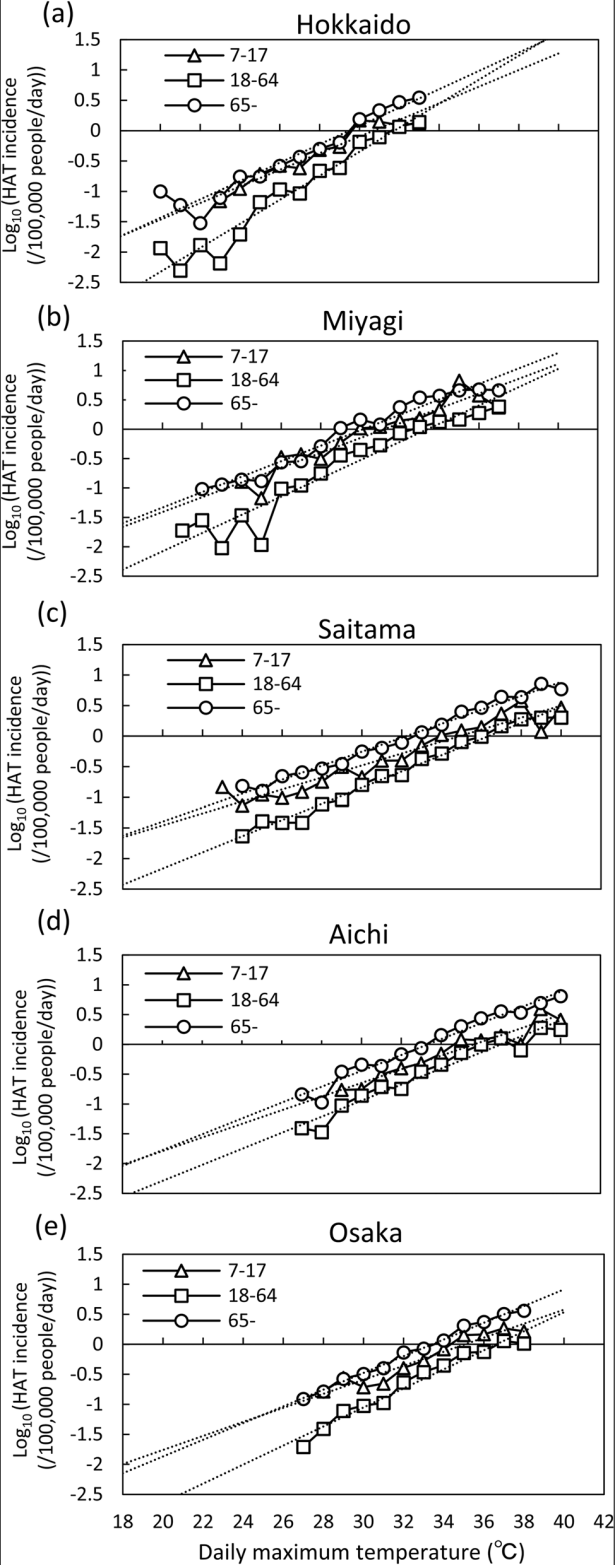
Incidence of HAT of each prefecture by age with DMT. *HAT* heat-related ambulance transport, *DMT* daily maximum temperature. **a** Hokkaido. **b** Miyagi. **c** Saitama. **d** Aichi. **e** Osaka

**Fig. 5:**
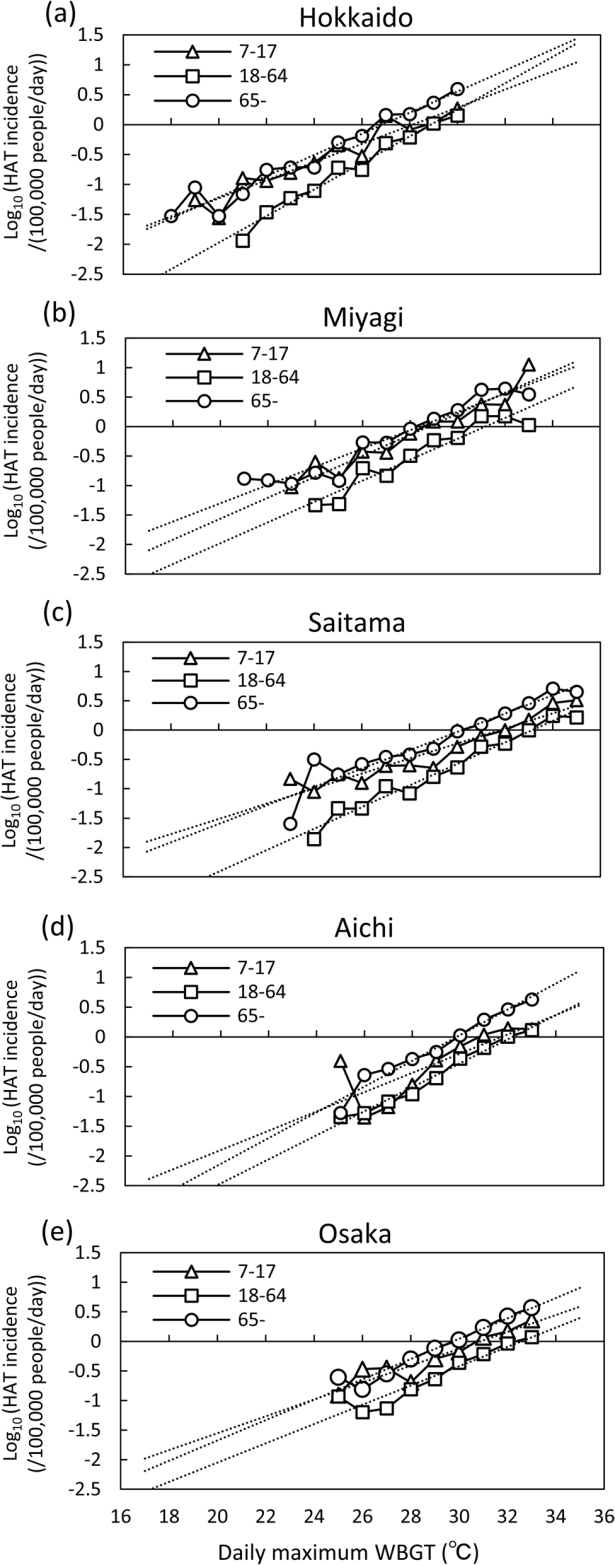
Incidence of HAT of each prefecture by age with DMW. *HAT* heat-related ambulance transport, *DMW* daily maximum WBGT. **a** Hokkaido. **b** Miyagi. **c** Saitama. **d** Aichi. **e** Osaka

Table 1 shows the mean, 3rd quartile and maximum DMT and HAT of age ranges from 7–17, 18–64, 65 years old and all ages for 47 prefectures and 10 regions with prefecture number, which corresponds with the number in Fig. 1. The DMT of 10 regions was obtain by averaging those of the prefectures belonging to each region weighted by their population. The logarithm of the incidence of HAT was plotted nearly on a regression line for DMT (Fig. 4) and DMW (Fig. 5) for each prefecture. In each prefecture, HAT was highest for elderly, and lowest for adults. The average (min–max) of *R*^2^ of the regression line relating logarithm of HAT to DMT for 47 prefectures were 0.82 (0.52–0.97), 0.91 (0.76–0.99), and 0.92 (0.79–0.99) for juveniles, adults, and elderly, respectively. The standard deviation of *R*^2^ of the regression line was 0.10, 0.06, and 0.05 for juveniles, adults, and elderly, respectively. The average slope of the regression line for 47 prefectures was 0.13, 0.15, and 0.14 (per degree) for juveniles, adults, and elderly, respectively.

**Fig. 6:**
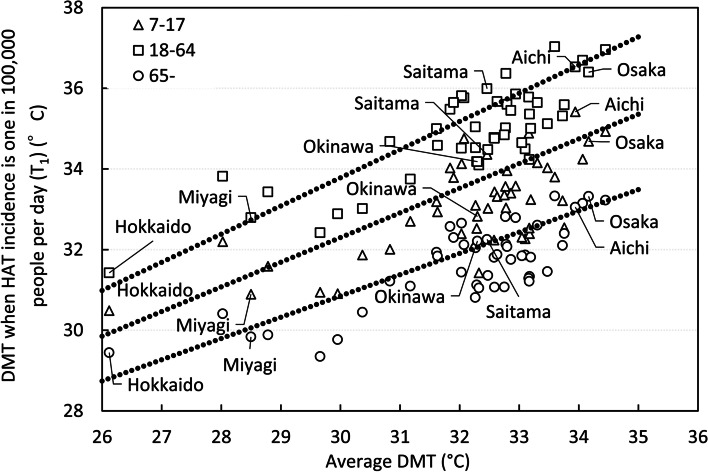
Relation between average DMT and T_1_. *HAT* heat-related ambulance transport, *DMT* daily maximum temperature. Average DMT is DMT averaged from July 21^st^ to the end of August for each year from 2017 to 2020. T_1_ is DMT that is predicted to cause HAT at a rate of 1 in 100,000 people per day. Each point represents the data of each prefecture

**Fig. 7:**
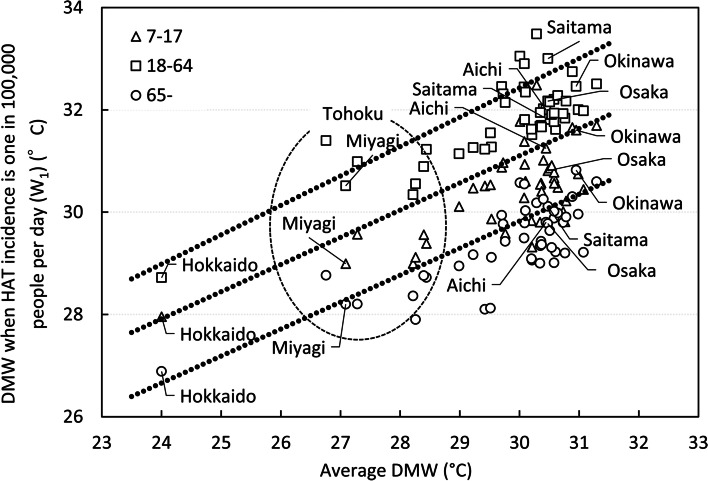
Relation between average DMW and W_1_. *DMW* daily maximum WBGT, *HAT* heat-related ambulance transport. Average DMW is DMW averaged from July 21st to the end of August for each year from 2017 to 2020. W_1_ is the DMW that is predicted to cause HAT at a rate of 1 in 100,000 people per day. Each point represents the data of each prefecture

The average (min–max) of *R*^2^ of the regression line for DMW were 0.86 (0.53–0.98), 0.93 (0.82–0.98), and 0.94 (0.82–0.99) for juveniles, adults and elderly, respectively. The standard deviation of *R*^2^ of the regression line was 0.09, 0.04, and 0.04 for juveniles, adults, and elderly, respectively. For the 47 prefectures, T_1_ and W_1_ were plotted against average DMT and DMW of each prefecture in Figs. 6 and 7, respectively. Average DMT and DMW were DMT and DMW averaged in T period. However, juveniles in Aomori and Okinawa prefecture, whose *R*^2^ values on the regression line relating HAT to DMW were exceptionally lower than 0.5 due to the exceptional emergency of HAT at low DMW, were excluded. Both T_1_ and W_1_ were the highest in adults and lowest in elderly. The regression line of Fig. 6 was drawn by the least square weighted by the population of each prefecture. *R*^2^ of the regression line of T_1_ to average DMT for adults was 0.77. Since the slope of regression line for adults in Fig. 6 was highest, T_1_ of adults was larger than elderly with a higher DMT in each prefecture. The slope of the regression line for 47 prefectures was 0.61, 0.70, and 0.53 (per degree) for juveniles, adults, and elderly, respectively.

The difference in average DMT between the highest DMT (Kyoto) (Fig. 1) and the lowest (Hokkaido) (Fig. 1) was 8.3 °C. The largest regional difference of T_1_ in the 47 prefectures was 4.9 °C for juveniles between Hokkaido and Aichi, 5.6 °C for adults between Hokkaido and Yamanashi, and 4.0 °C for elderly between Fukushima and Yamanashi. Concerning age differences, in a prefecture where the average DMT was 32 °C, T_1_ was 33.5 °C, 35.2 °C and 31.9 °C for juveniles, adults, and elderly, respectively (Fig. 6). Similarly, the difference in DMW between the largest DMW (Nagasaki) (Fig. 1) and lowest DMW (Hokkaido) was 7.3 °C. The largest regional difference of W_1_ in 47 prefectures was 4.5 and 4.8 °C for juveniles and adults, respectively between Hokkaido and Tokyo, 3.9 °C for elderly between Hokkaido and Okinawa. The regression line of Fig. 7 was drawn by the least square weighted by the population of each prefecture. For age differences, in a prefecture where the average DMW in T period was 30 °C, W_1_ was 31.1 °C, 32.4 °C, and 29.8 °C for juveniles, adults, and elderly, respectively (Fig. 7).

## Discussion

Two-way ANOVA showed that region and age significantly affected HAT occurrence (Fig. 2). The logarithm of HAT by prefecture and age class fitted well to the linear function of DMT or DMW. Moreover, T_1_ and W_1_ of each prefecture was approximated well by a straight line against the average DMT and DMW in T period of each prefecture. The maximum regional differences of T_1_ and W_1_ for adults in Japan were 5.6 and 4.8 °C, respectively. HAT occurrence was affected by age. In a prefecture with a mean DMT of 32 °C, T_1_ was 33.5, 35.2, and 31.9 °C and in a prefecture with a mean DMW of 30 °C, W_1_ was 31.1, 32.4, and 29.8 °C for juveniles, adults, and elderly, respectively.

Previous studies [19–22] have also shown that there is an age difference in the incidence of heat stroke. The relative ratio of HAT during a heat wave compared with usual days increased by two times in the 65- age group compared with the 5–64 age group [19]. The impact of a heat wave on heat-related illness would remarkably appear because HAT during a heat wave was short-term. However, a heat-related illness study in North Carolina from 2007 to 2012 showed that HAT per unit person-years of the 18–44 age group was the largest in five age groups and that of the 65– age group was the fourth group [20]. HAT data from the CDC [21] showed that HAT incidence per 1000 person-years of 5–14, 15–34, 35–64, and 65– were 0.170, 0.419, 0.324, 0.377, respectively. Compared with the results of our study, the HAT incidence for 18-64 was the same, but that for 65- was about 2.7 times larger than CDC data. A combination of environmental and socioeconomic risk factors, such as home air conditioning, housing and urban design, and social isolation could affect the occurrence of HAT.

T_1_ in a prefecture where the average DMT in T period is 32.0 °C was estimated to be 33.5, 35.2, and 31.9 °C for juveniles, adults, and elderly, respectively. This result means that elderly people reached a HAT incidence of one in 100,000 people per day at a 3.3 °C lower DMT than that of adults. According to our previous study about HAT in Japan [22], the HAT incidence per unit people did not change significantly from those in their 20s to those in their 60s, while for those above their 70s, the increase in heat stroke became remarkable. Therefore, HAT is expected to increase with age for those aged 65 and older.

Various physiological factors are involved in the susceptibility of the elderly to heat stroke [23]. The body's response to heat dissipation from the body is mainly through peripheral cutaneous vasodilation and the sweating function, but these two functions are reduced in the elderly. Due to a reduced pumping function of the heart and decreased amount of water in the body, the elderly is not able to pump a large amount of blood to the periphery during heat stress, and consequently the amount of sweating decreases. Young people are superior in the heat dissipation function during exercise due to a larger amount of sweating than the elderly [24]. In addition, since the sense of heat is reduced in the elderly, it is difficult to invoke behavioral thermoregulation, such as turning on an air conditioner [25]. The majority of HRM for elderly occur at home [26]. The heat wave that hit Europe in 2003 resulted in a high incidence of heat stroke deaths in residential homes in France, with a 1.9-fold increase in deaths among people aged 75 and older [26]. During heat waves, indoor heat levels of many low- and middle- income New York City households regularly reach dangerous heat levels for human health [27].

Most HAT of juveniles occurred during exercise at school. Since considerable heat is produced in the body during exercise, body temperature rises. Moreover, the amount of heat dissipation is limited because the sweat function is still immature before puberty. Around puberty, the sweat rate increases significantly [28]. Therefore, juveniles are more likely to suffer from HRI than adults and develop HRI at a relatively low temperature. This epidemiological study also showed that T_1_ of juveniles was 1.7 °C lower than that of adults in a province with an average DMT of 32 °C.

**Fig. 8:**
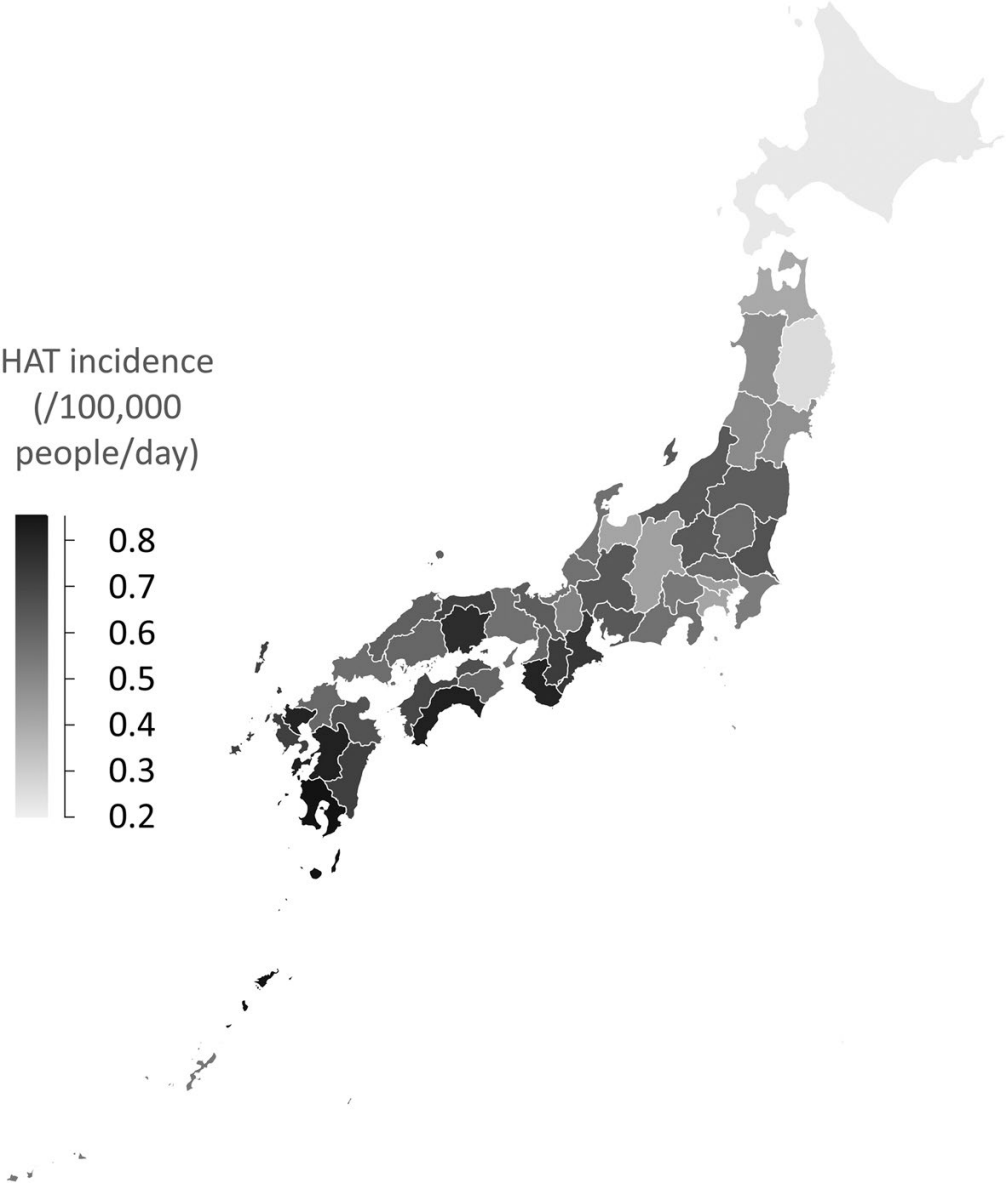
HAT incidence for 18-64 years old by each prefecture. *HAT* heat-related ambulance transport

**Fig. 9:**
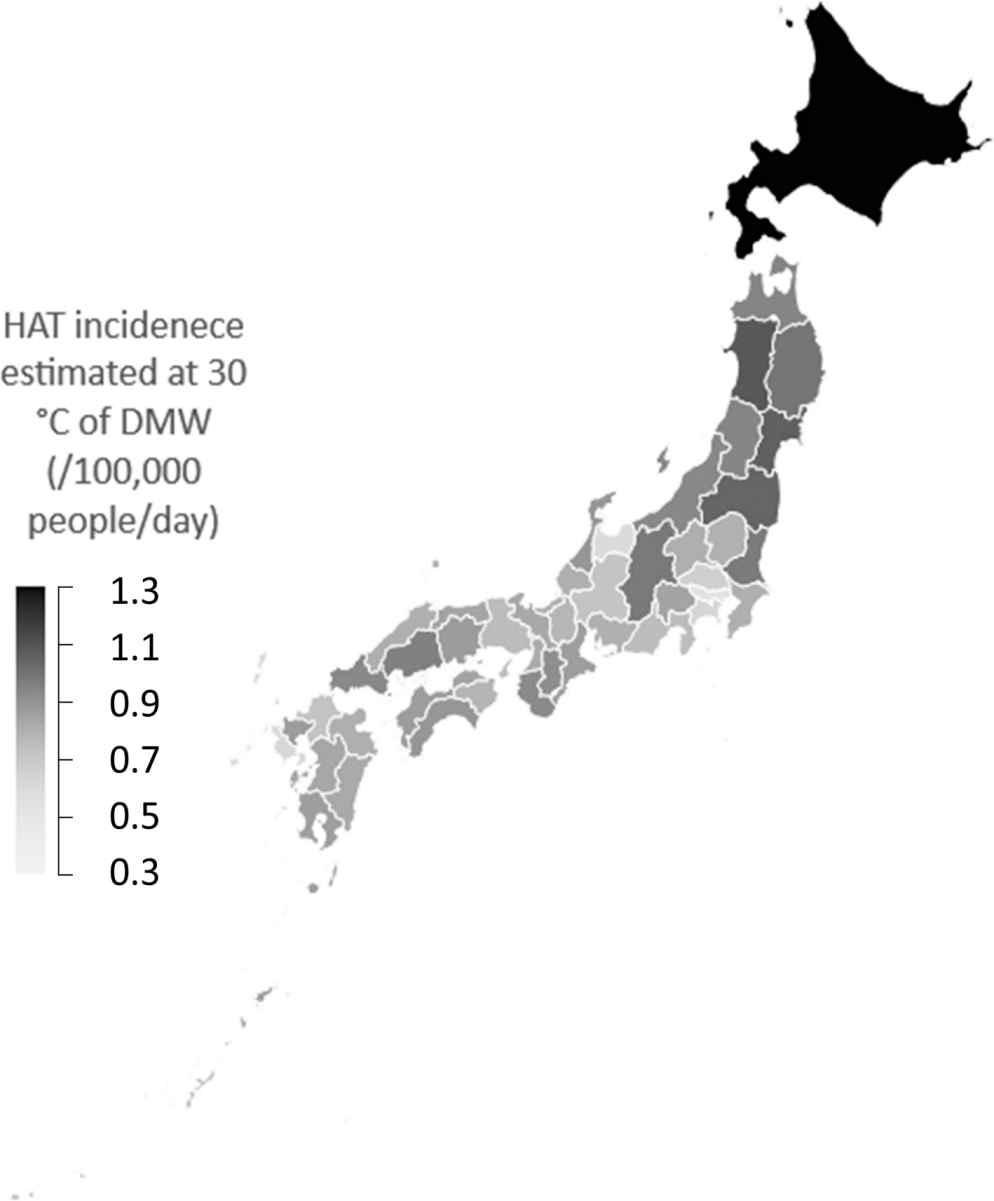
HAT incidence estimated at 30 °C of DMW for 18–64 years old by each prefecture. *HAT* heat-related ambulance transport

A regional difference study of HRM in Japan [9] showed that HRM tended to be higher in the inland areas of the Kanto Region and on the Sea of Japan side, and lower on the Pacific side. In this study, HAT tended to be higher in western Japan, but there were no differences between the Sea of Japan side and the Pacific side. HAT tended to increase in southern regions but decreased in the southernmost prefecture of Okinawa (Fig. 3). It is speculated that HAT decrease in Okinawa, being an island and surrounded by the sea, would be caused by little fluctuation in temperature and few extremely hot days. Table 1 shows that maximum DMT and 3rd quartile DMT are lower than the average of 47 prefectures. Figure 8 shows HAT incidence of adults for 47 prefectures. In the Kyushu and the Shikoku Region, HAT incidence was higher than in Hokkaido and the Tohoku Region because the summer climate in the southwest was severe (Table 1). Fujibe [29] reported that HAT incidence of each prefecture was positively correlated with the average DMT of each prefecture, which showed that outdoor temperature had a strong effect on the occurrence of heat-related illness. HAT incidence is affected by both weather conditions in the summer and the susceptibility of the population to heat. To eliminate the effect of weather condition differences in each prefecture for HAT, assuming that the DMW of all prefectures is at a constant of 30 °C for DMW, the estimated HAT incidence for adults determined by using a linear regression line for each prefecture (Fig. 4) is shown in Fig. 9. Contrary to Fig. 8, HAT incidence was highest for Hokkaido, followed by the Tohoku Region in Fig. 9. Figure 9 shows that in cool summer regions, people are less acclimatized to heat than in hot regions. Fujibe [29] also showed that at the same outdoor temperature, HAT incidence of each prefecture decreased with an increase of average DMT in summer. This property is shown in Figs. 6 or 7 in our study. The vertical axis of T_1_ or W_1_, which indicate less susceptibility to heat stroke of the people in the prefecture, increases as the average DMT in T period increases (Figs. 6 and 7). As to the originality of our study, we indicated that the units of T_1_ and W_1_, which indicate susceptibility to heat stroke, are temperature and WBGT, respectively. Thus, it can be used as a reference when formulating environmental standards for heat stroke that takes regional and age differences into consideration.

People become acclimatized to heat by exposure to local weather conditions. However, acclimatization is not a binary characteristic. The heat acclimatization level varies from prefecture to prefecture. Partial acclimatization occurs depending on the season or the climate of the place of residence [30]. The frequency of going out also affects the level of heat acclimatization. Even when persons live in the same area, the level of heat acclimatization of those working outdoors is higher than those who rarely go outdoors. Moreover, people in mild summer regions are not prepared in advance to avoid heat-related illness due to a lack of experience [30], and accordingly the estimated HAT incidence is higher than in hot regions (Fig. 9).

In a laboratory study, with several hours of heat exposure per day, heat acclimatization is completed within two weeks [31]. In the early summer, when the period of acclimatization is not so long, people are in a partially acclimatized state. Also in the late summer, heat acclimatization begins to disappear as the harsh weather ends. In this study, only the data of HAT from the end of July to the end of August was analyzed, when people were expected to be most acclimatized to heat.

To investigate heat acclimatization that occurs in daily life, seasonal differences in the amount of sweating or salinity concentration in sweat have been measured in subject experiments. In Kyoto (Fig. 1) in the Kinki Region [32], the same young man was exposed to heat every two months and the amount of sweating was measured. The amount of sweating in August was twice as much as the other months. In a subject experiment conducted in Aichi (Fig. 1) in the Tokai Region, the chlorine concentration in sweat decreased from May, reached a minimum in August, and increased from autumn [33]. In addition, in a test conducted in Kobe (Fig. 1) in the Kinki Region showed that both young people (22 years old on average) and elderly (62 years old on average) sweated more in the summer and the sodium concentration in sweat was low in summer, indicating the effect of heat acclimatization [25]. In comparison, in an experiment with subjects conducted in Ottawa Canada, the amount of sweating, skin blood flow, core body temperature, and average skin temperature did not differ between the latter half of May and the first half of September [34], indicating no heat acclimatization in the summer. In Ottawa, the maximum daily temperature in August was around 25 °C. Since the summer climate in Hokkaido is almost the same as Ottawa, it is presumed that the heat acclimatization there in summer is also small. Another possible reason why DMT or DMW when HAT occurs is relatively low in Hokkaido is that preventive measures against heat stress are not thoroughly done because there are not many opportunities to be exposed to heat in Hokkaido. Previous studies have shown that there are regional differences in the incidence of morbidity [8, 18, 21, 35] and mortality [1, 2, 7, 9, 36–38]. Every study pointed to a higher risk in summertime morbidity or mortality in cooler than warmer regions. It has been reported that the number of HAT for heat stroke increases sharply when there are exceptionally hot days in cool summer regions [35].

The north-south difference of T_1_ in Japan was 5.6 °C, and the W_1_ difference was 4.8 °C for adults. These results show that public health policies to prevent HRI should be tailored according to the local climate. However, even ISO7243 and ACGIH-TLV, which are international representative standards for heat stroke, only target healthy and physically suitable workers, and do not take regional or age differences into account. Grundstein [39] has proposed a heat standard that considers the level of heat acclimatization of each region. In the USA, the 90th percentile of the DMW in summer varies from 36 °C or higher to 26 °C or lower depending on each region, so heat acclimatization also differs. By dividing the USA into three categories, different heat guidelines were proposed for sports during hot weather. In Japan, the average DMW by prefecture differs by 7.3 °C from the maximum to the minimum, so it would be desirable to consider whether different heat standards for different regions are necessary.

The strength of this study is that the effects of age and region on HAT were converted into the temperature difference at which HAT occurs. In the analysis process of HAT, some simplifications are made. First, we did not consider the delayed effect of high temperature on HAT, but only DMT or DMW on the same day of HAT. Previous studies have shown that DMT on the same day had the strongest effects on HAT [40]. The major features of HAT were extracted from the data. Subsequently, the analyzed DMT or DMW was not the temperature of the place where HAT occurred. We hypothesized that outdoor weather conditions affected how heat stroke developed. In the elderly, people with illnesses such as circulatory diseases often develop heat stroke indoors. The temperature inside a closed room unequipped with a cooler could be higher than outside. Moreover, even if the location of HAT is outdoors, the temperature of the place of occurrence does not always correspond to the temperature measured at a weather station. To reduce the temperature error as much as possible, the population-weighted DMT or DMW measured at three or six weather stations in each prefecture was used. In this study, the exponential function was assigned to the relationship between DMT or DMW and HAT. The relationship between the logarithm of the incidence of HAT and DMT or DMW was expressed as an almost linear relationship in terms of high mean *R*^2^ of the regression lines. Some *R*^2^ value were about 0.5 for juveniles due to the small number of HAT occurrences at relatively low temperatures in a prefecture with a small population. The slope of regression lines (Figs. 4 or 5) indicates how many times HAT increases as DMT or DMW rises by 1 °C. Previous studies [18] about HAT in Japan showed that an exponential function was very applicable to the relationship between DMT and HAT. As a result, this study also showed that an exponential function was suitable as a fitting function between DMT and HAT.

## Conclusion

The present study showed that age and local climate affects heat-related ambulance transports (HAT) from July 21st to the end of August for each year from 2017 to 2020 (T period). Annual incidences per 1000 persons of HAT from July 21st to the end of August were 0.41, 0.23, and 0.65 for juveniles, adults, and elderly, respectively. The logarithm of the incidence of HAT was plotted nearly on a regression line for daily maximum temperature (DMT) and daily maximum WBGT (DWT) for each prefecture and for three age categories. The average and range of *R*^2^ of the regression line for DMW of 47 prefectures were 0.86 (0.53–0.98), 0.93 (0.82–0.98), and 0.94 (0.82–0.99) for juveniles, adults and elderly. DMT or DMW expected when the daily incidence of HAT was one in 100,000 people, T_1_ or W_1_, was calculated as an indicator of heat acclimatization of local people. The largest regional difference of W_1_ in 47 prefectures was 4.5 and 4.8 °C for juveniles and adults, respectively between Hokkaido and Tokyo, 3.9 °C for elderly between Hokkaido and Okinawa. For age differences, in a prefecture where the average DMW in T period was 30 °C, W_1_ was 31.1 °C, 32.4 °C, and 29.8 °C for juveniles, adults, and elderly, respectively. Our results indicate that public prevention measures for heat-related disorders, such as heat stroke advisories issued from public agencies, should take into consideration the various characteristics of HAT due to age and local area.

## Abbreviations

HAT: Heat-related ambulance transport
DMT: Daily maximum temperature
WBGT: Wet bulb globe temperature
DMW: Daily maximum WBGT
HRM: Heat-related mortalities
HRI: Heat-related illness
